# Disease and Crime

**Published:** 1857-01

**Authors:** 


### HALL'S JOURNAL OF HEALTH.

OUR LEGITIMATE SCOPE IS ALMOST BOUNDLESS: FOR WHATEVER BEGETS PLEASURABLE AND HARMLESS FEELINGS, PROMOTES HEALTH; AND WHATEVER INDUCES DISAGREEABLE SENSATIONS, ENGENDERS DISEASE.

VOL. IV.] JANUARY, 1857. [NO. I.

We aim to show how disease may be avoided, and that it is best, when sickness comes, to take no medicine without consulting an educated physician.

### DISEASE AND CRIME.

LIGHT is daily coming in upon the world of mind, and by the help of clearly established facts, arguments may be adduced, which will have a stronger tendency to compel men to take care of their health, than any which have arisen from conscience, money or duty; that is, the argument of *Shame.* Let men fully understand that certain bodily affections tend to crime, and that crime thus committed confines to the Penitentiary, then may the community wake up more fully to the sentiment,

HEALTH IS A DUTY, and therefore, the neglect of its preservation, a sin, which in the natural progress of things, leads to loss of health, and life, and honor.

In a recent trial of a forger, who handled millions of dollars in a year's business, the defence was that he was insane. Among the evidence offered was that he could sleep only three or four hours out of the twenty-four. In a previous number we stated, that a growing inability to sleep was a clear indication of approaching insanity, and on the return of sleepfulness, the intellect became clear. There were other symptoms. There was the sound of trip-hammers in his ears; blacksmith's sparks floated before his eyes, and there was pain in the head a large portion of the time. These symptoms, lasting so long, had at length so affected the brain, as to destroy all perception, or comprehension of the *effects* of crime; and when the organ of a man's perception is destroyed, he will plunge headlong, and with utter recklessness, into any kind of wrong-doing which circumstances throw in his way—-arson, robbery, murder, anything; and, if not detected or prevented, the crime, whatever it may be, will grow into a habit, and habit is second nature; consequently, he will revel in it, it becomes his meat and drink, and he would rather do it than not. Hence the prisoner declared without hesitation, that if he were released he would do it again; that he rather liked it, and nothing could prevent him but cutting off his hand, if it came in the way, to forge paper.

It was shown on the trial, that there was insanity on the father's and mother's side; but no indication of it on the part of either father or mother. It is well known however, that insanity, as well as personal features, overleaps a generation or two. Often a child bears a striking resemblance to a grand-parent, without a lineament of parental feature.

The acts of the prisoner were admitted by his counsel, and the question of guilt or innocence, rested on this—was he insane or not?

The use which we wish to make of these developments is practical, and is of high importance. A wise and stern medical treatment would have deferred, if not prevented, the combination of events. And how?

The prisoner was under the habitual influence of constipation, and an anodyne, which intensified this constipation every hour, while the principle of the medical practice in this case, was to let the bowels take care of themselves—which they did not do. This individual was never seen by his business associates without a cigar in his mouth; he smoked fifteen or twenty a day. The immediate effect of smoking tobacco falls on the brain, excites it; during that excitement he could not sleep, and the reaction went so low that he could not sleep; only a troubled repose was possible during the brief transition from one to the other. During the excitement, the brain ran riot in the direction of the opportunity, and expended its energies in that direction, but during the reaction, power was not left to carry on the bodily functions.

The effect of constipation is to thicken the blood, to make it more impure: hence more unfit for healthful purposes. The more impure the blood is, the thicker does it become, the slower is its progress, and if nothing is done to alter this state of things, stagnation and death take place. Stagnation means accumulation, for the moment the blood stops in any part of the body, the coming current flowing in, causes an accumulation, precisely as in the closing of a canal gate, or the damming up of a stream. This accumulation in the blood vessels distends them, causes them to occupy more room than nature designed, consequently they must encroach on their neighbors. The neighbors of the blood vessels are the nerves; hence the nerves are pressed against; that pressure gives what we call "pain." As there are nerves everywhere, a point of a needle cannot be placed against the surface of the body without some pain, which shows the universality of nerve presence; hence, we may have pain anywhere, and will have pain if there is pressure. This accounts for the steady pain in the head. The excitement of the day sent the blood to the brain too fast, the repose of the night was too short to allow of its removal; besides the energies of the system had been overtaxed, and there was not power enough left to remove a natural accumulation, let alone the extraordinary.

But there is a law of our body, whereby pressure from any cause not only gives pain, but may destroy the part pressed against, and consume it, by dissolving it into a gaseous and fluid substance, which in this condition is conveyed out of the body. A band put around an arm of a foot in circumference, will, if tightened every day, in a time not long, reduce the circumference to six inches. Constant pressure cannot be exerted against any portion of the human body without impairing its structure, or causing its diminution and final destruction. These are principles of universal admission. They are first truths in medicine. From some *unknown* cause, this accumulation and pressure was determined to a particular portion of the brain, where fearlessness of consequences are situated; and we believe, if the prisoner's brain could be examined this day, that portion of it, most probably small in the beginning, would be found almost wholly wanting, having been destroyed by long continued pressure, *or* to be of abnormal structure.

We believe that a medical treatment, which would have sternly interdicted the use of the cigar materially at first, and gradually thereafter, until its final extinction, together with securing a natural condition of daily acting bowels, with a plain and substantial diet—*and kept him there*—would have saved him and all his from the subsequent calamities. Artificial excitements, whether from tobacco, opium, or alcohol, if largely persevered in, will work ruin to mind, body, and soul. It is right that it should be so. Omnipotence has ordained it. If a man is in a physical condition which impels him to do what is illegal, or if he be in a mental condition which impels him to do what is illegal, the question whether he is to be punished or not depends upon the manner in which he became subjected to that condition. If such condition be the result of birth, or by a fall, or stroke, or other occurrence out of his control, he should go free of penal suffering; but if he placed himself in that condition by the unbridled indulgence of his appetites or his passions, he ought to be made to suffer a just penalty, whether he knew that such indulgences tended to such a result or not. It is a man's duty to inform himself of physiological as well as civil law. Ignorance of the former ought not to work his escape, any more than ignorance of the latter does; otherwise, a man has only to get drunk to secure impunity from any crime which may be committed in that condition; thus all penal statutes become a farce, and anarchy rides rampant through the land.

So also, if a man perverts his moral sense, and by a course of vicious reasoning persuades himself that he ought to commit murder, and thinks of it so much as to feel impelled to murder some one, he is properly amenable to the law of the land.

It is no very difficult matter for ordinary minds to persuade themselves as to any desired course—that it is right; that there is no harm in it; and that, if they meant no harm by it, no blame could be attached; but, if for such flimsy considerations, men are to be excused from penalties, there is an end at once to all law and to all government.

The conclusion of the whole matter is this. Every man should be held responsible for his deeds, unless they are clearly proved to be the result of a physical, mental, or moral condition which he had no agency in originating, or exaggerating to the criminal point. Hence the prisoner was convicted.

